# Residential crowding and severe respiratory syncytial virus disease among infants and young children: A systematic literature review

**DOI:** 10.1186/1471-2334-12-95

**Published:** 2012-04-20

**Authors:** Ann D Colosia, Anthony Masaquel, Caroline Breese Hall, Amy M Barrett, Parthiv J Mahadevia, Ram Yogev

**Affiliations:** 1 RTI Health Solutions, Research Triangle Park, NC, USA; 2 MedImmune, Gaithersburg, MD, USA; 3 University of Rochester Medical Center, Rochester, NY, USA; 4 Ann and Robert Laurie Children’s Hospital of Chicago, Feinberg School of Medicine, Northwestern University, Chicago, IL, USA

**Keywords:** Respiratory syncytial virus, Respiratory infection, Crowding, Children

## Abstract

**Background:**

The objective of this literature review was to determine whether crowding in the home is associated with an increased risk of severe respiratory syncytial virus (RSV) disease in children younger than 5 years.

**Methods:**

A computerized literature search of PubMed and EMBASE was conducted on residential crowding as a risk factor for laboratory-confirmed RSV illness in children younger than 5 years. Study populations were stratified by high-risk populations, defined by prematurity, chronic lung disease of prematurity, hemodynamically significant congenital heart disease, or specific at-risk ethnicity (i.e. Alaska Native, Inuit), and mixed-risk populations, including general populations of mostly healthy children. The search was conducted for articles published from January 1, 1985, to October 8, 2009, and was limited to studies reported in English. To avoid indexing bias in the computerized databases, the search included terms for multivariate analysis and risk factors to identify studies in which residential crowding was evaluated but was not significant. Methodological quality of included studies was assessed using a Cochrane risk of bias tool.

**Results:**

The search identified 20 relevant studies that were conducted in geographically diverse locations. Among studies of patients in high-risk populations, 7 of 9 found a statistically significant association with a crowding variable; in studies in mixed-risk populations, 9 of 11 found a significant association with a crowding variable. In studies of high-risk children, residential crowding significantly increased the odds of laboratory-confirmed RSV hospitalization (i.e. odds ratio ranged from 1.45 to 2.85). In studies of mixed-risk populations, the adjusted odds ratios ranged from 1.23 to 9.1. The findings on the effect of residential crowding on outpatient RSV lower respiratory tract infection were inconsistent.

**Conclusions:**

Residential crowding was associated with an increased risk of laboratory-confirmed RSV hospitalization among high-risk infants and young children. This association was consistent despite differences in definitions of residential crowding, populations, or geographic locations.

## Background

Almost all children contract respiratory syncytial virus (RSV) in the first two years of life, and lower respiratory tract illness is most likely to occur during the first RSV infection [1]. Although most of these RSV infections are mild, RSV can produce serious illness requiring hospitalization, especially among high-risk infants [2]. RSV has been recognized throughout the world to cause a major proportion of pediatric hospitalizations and pneumonia [3]. In the United States (US) during the fall and winter seasons, RSV accounts for approximately 30–90% of bronchiolitis and up to 50% of hospitalizations for pneumonia among infants [4]. The average annual hospitalization rate is 3 per 1,000 children younger than 5 years and 17 per 1,000 children younger than 6 months [2]. Across Europe, illness diagnosed as being due to RSV accounts for 42–45% of hospitalizations for lower respiratory tract infection (LRTI) in children younger than 2 years [5]. The proportion of laboratory-confirmed RSV infection among young children hospitalized for acute LRTI was 29% in Brazil [6], 36% in China [7], and 40% in Australia [8]. Studies in Brazil [6] and Australia [8] also found that 60% and 63%, respectively, of infants hospitalized with bronchiolitis were RSV positive. Data for the developing world are limited, but studies from Kenya [9] and western Gambia [10] found that 11% and 19%, respectively, of young children with serious LRTI had laboratory-confirmed RSV infection.

The American Academy of Pediatrics’ Committee on Infectious Diseases has issued recommendations regarding prevention of severe RSV disease among high-risk infants and children [1]. For children born prematurely (32 weeks 0 days to 34 weeks 6 days gestation), having at least one of two risk factors—day care attendance or having a sibling younger than 5 years living permanently in the household—warrants consideration for prophylaxis against severe RSV disease with palivizumab, a monoclonal antibody with neutralizing and fusion-inhibitory activity against RSV. Day care attendance and having young siblings were considered potential risk factors because a number of studies have shown that these factors were associated with an increased likelihood of severe RSV disease [1]. Both of these risk factors bring an at-risk child into close proximity with potentially infected individuals. Development of infection within a home may be enhanced by greater crowding, which increases the likelihood of exposure to droplets of infectious secretions and of self-inoculation after contacting fomites or surfaces contaminated with RSV [11,12]. This systematic literature review, therefore, explored the association of residential crowding and the risk of severe laboratory-confirmed RSV disease among young children younger than 5 years.

## Methods

### Patients

A systematic literature search was conducted for studies assessing the association of residential crowding with severe RSV disease in children younger than 5 years, including populations considered at high risk of developing severe RSV disease.

High-risk populations were defined as premature infants, children with chronic lung disease (CLD) of prematurity or hemodynamically significant congenital heart disease, or members of an ethnic group believed to be at increased risk of respiratory disease (i.e. Alaska native, Inuit). Other study populations were defined as mixed-risk and consisted predominantly of children from the general population not meeting the high-risk definition. Children in the mixed-risk population may have had varying levels of risk because of other factors that increase the risk of severe RSV disease, such as young age (≤1 year) [2]. Studies limited to populations with severe health conditions, such as cancer or organ transplantation were excluded.

### Data sources and search methods

The literature search included studies published and indexed in the PubMed (including MEDLINE) and EMBASE databases between January 1, 1985, and October 8, 2009 (see Additional file 1 for the search strategies used for each database). The PubMed search used the National Library of Medicine medical subject heading (MeSH) terms “respiratory syncytial virus infections” or “bronchiolitis, viral” or “respiratory tract infections” combined with the words “syncytial” or “RSV” or “acute” plus “lower” anywhere in the database record. To focus the search on studies evaluating risk factors, MeSH terms were limited to the subheadings “epidemiology,” “etiology,” and “complications.” To identify studies of residential crowding as a risk factor for severe RSV disease, the search used the MeSH term “crowding” or any of the following words describing crowding: “resident,” “residents,” “residential,” “bedroom,” “bedrooms,” “household,” “households,” “number of children,” “number of people,” “per room,” “> and people,” “sibling,” “siblings,” “sharing,” “share,” or “overcrowding.”

Studies that examined multiple risk factors for severe RSV disease were also included in the full-text review, whether or not residential crowding variables were mentioned in the abstract. This approach ensured that the present study avoided the bias caused by overlooking nonsignificant findings related to crowding (which may not be highlighted in a study’s abstract when a set of risk factors are examined). To identify these studies, titles or abstracts were searched for a respiratory disease–related term, as described above, and any of the text words describing analysis of multiple risk factors: “multivariate,” “multifactorial,” or “regression.” This broader search was limited to the population of interest by also requiring use of any of the words “child,” “children,” “infant,” or “infants.”

### Study selection and data extraction

**Figure 1:**
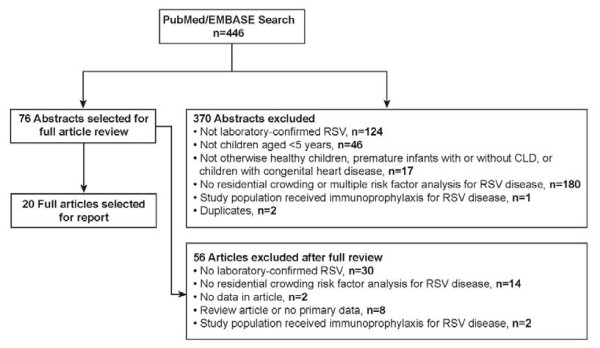
**Flow diagram of inclusions and exclusions.** CLD = chronic lung disease; RSV = respiratory syncytial virus.

The titles and abstracts of articles published in English and identified in the database searches were reviewed for potential relevance (Figure 1). For relevant sources, the full articles were obtained and reviewed to determine whether residential crowding was assessed. Only studies in which the children had lower respiratory tract illness due to laboratory-confirmed RSV and were younger than 5 years were included. Any treatment setting for RSV was accepted; however, the majority of the studies were on populations hospitalized with an RSV infection. Few studies examined patients with RSV lower respiratory tract infection in an outpatient setting. In contrast to RSV hospitalization, outpatient RSV illness reflects mild disease. Each relevant article was read by several authors. Study details were extracted into tables by one author, and the content of the tables was verified by a second author not involved in the data extraction. The authors discussed each article to reach consensus regarding the study details. For each study, the following data were extracted: reference, publication year, country origin, study design, study population size and description, residential crowding definition and categories, disease outcome evaluated, results and significance of unadjusted analyses, adjusted analyses, and other statistical analyses. The principal summary measures were adjusted odds ratio (aOR) or adjusted relative risk (aRR) for multivariate analyses and odds ratio (OR) for bivariate analyses.

### Bias assessment

The risk of bias was assessed using the Cochrane risk of bias tool [13]. The assessment focused on likely sources of bias in observational studies. The reviewed studies were examined for risk of selective reporting bias and confounding bias that pertained to the association between residential crowding and RSV disease. Selective reporting bias can occur if outcomes for all studied risk factors are not reported. Not reporting outcomes of all variables could bias the reader’s interpretation of the outcomes. For example, reporting only outcomes for a measure of crowding that was significant but not reporting the negative outcomes of alternative definitions of crowding could overstate the importance of residential crowding. Alternatively, reporting only negative outcomes of a single crowding variable in multivariate analysis, but not the positive associations of numerous measures of crowding in unadjusted analysis, may obscure potential explanations for the negative finding. Confounding bias may have been introduced if a study did not control for the effects of other variables in multivariate analysis and may lead to either over- or underestimation of the impact of residential crowding on risk of severe RSV disease, depending on how correction for other variables affects the outcome with the crowding variable. A more detailed explanation regarding bias assessment is presented in Additional file 2.

## Results

The search yielded 446 unique articles. The initial review of titles and abstracts identified 76 articles appropriate for full review. The full review identified 20 studies that met the inclusion criteria of analyzing the association of residential crowding with the risk of laboratory-confirmed RSV infection in children younger than 5 years (Figure 1).

### Overview of studies

Table 1 provides an overview of these 20 studies, including whether they reported a significant association between RSV disease and a residential crowding variable in the adjusted or unadjusted analysis. The studies were grouped by the risk status of the study populations: 9 studies were conducted in high–risk children, and 11 studies were conducted in populations with mixed risk. Most of the studies examined laboratory-confirmed RSV hospitalization; a few studies based their analyses on laboratory-confirmed RSV infection diagnosed or treated in the emergency department [14], outpatient clinic [9], combination of hospital and outpatient settings [15], or pediatrician’s office [16,17]. All of the studies were observational. Most of the studies were large, having hundreds or in some cases thousands [18-20] of subjects, although a couple of studies were small [21,22]. The studied populations were geographically diverse.

**Table 1:** **Overview of studies of residential crowding and RSV disease by risk status**^**a**^**(N = 20)**

				**Crowding Variables**		
**Reference**	**Study Type/Population**	**Numberof Participants**	**Age Criteria**	**≥1 Significant in Unadjusted Analysis**	**≥1 Significant in Adjusted Analysis**	**Bias Assessment**^**b**^
*High-risk population, (n = 9)*						
**Broughton et al., 2005**^**c**^[15]	Cohor, <32-week GA, UK	N = 126	≤1 year	Not conducted	Yes	No apparent bias concerns
**Bulkow et al., 2002**[23]	Case–control, Alaska natives, US	Cases n = 204 Controls n = 338	<3 years	Yes	Yes	No apparent bias concerns
**Carbonell-Estrany et al., 2000**[24]	Cohort, ≤32-week GA, Spain	N = 584	≤1 year	Yes	Yes	No apparent bias concerns
**Carbonell-Estrany et al., 2001**[25]	Cohort, ≤32-week GA, Spain	N = 999	≤6 months in October	Yes	Yes	No apparent bias concerns
**Figueras-Aloy et al., 2004**[26]	Case–control, 33- to 35-week GA, Spain	Cases n = 186 Controls n = 371	<1 year	Yes	Yes	No apparent bias concerns
**Figueras-Aloy et al., 2008**[18]	2 Cohorts, 32- to 35-week GA, Spain	Cohort 1 (cases), n = 202 Cohort 2 (controls), n = 5239	Discharged during or ≤6 months of age at start of RSV season	Yes	Not applicable^d^	No apparent bias concerns
**Kanra et al., 2005**[27]	Cohort, 64.3% preterm (<35-week GA), 20.8% CHF, Turkey	N = 332	<6 months and preterm; <2 years with CLD	No	Not conducted	No multivariate analysis
**Law et al., 2004**[19]	Cohort, 33- to 35-week GA, Canada	N = 1860	≤7 months (or older to end of RSV season)	Yes	Yes	No apparent bias concerns
**Simoes et al., 1993**[22]	Case–control, Multiples vs. singletons, all ≤32-week GA with CLD; US	Cases n = 34 Controls n = 34 Combined group analyzed	<2 years	Yes	NR	Multivariate analysis NR
*Mixed-risk population, (n = 11)*						
**Albargish and Hasony, 1999**^**c**^[16]	Cross-sectional study, Iraq	n = 500 with LRTI (37.6% RSV-positive); n = 57 controls	<5 years	No	Not conducted	No multivariate analysis
**Flores et al., 2004**^**c**^[14]	Cohort, Portugal	N = 225 (137 RSV-positive, 88 RSV-negative)	<3 years	No	Not conducted	No multivariate analysis
**Hayes et al., 1989**[21]	Case–control, American Samoa	Cases n = 13 Controls n = 45	<2 years	Yes	Not conducted	No multivariate analysis
**Holberg et al., 1991**^**c**^[17]	Cohort, US	N = 1179	≤1 year	Yes	Yes	No apparent bias concerns
**Lanari et al., 2002**[28]	Cohort, Italy	N = 1232	<2 years	Yes	Not conducted	No multivariate analysis
**Nielsen et al., 2003**[20]	Case–control, Denmark	Cases n = 1272 Controls n = 6075	<2 years	Not conducted	Yes	No apparent bias concerns
**Okiro et al., 2008**^**c**^[9]	Cohort, Kenya	N = 469	<2 weeks old, followed for 3 RSV seasons	Yes	Yes	No apparent bias concerns
**Reeve et al., 2006**[29]	Case–control, Australia	Cases n = 271 Controls n = 542	<3 years	Not conducted	Yes	No apparent bias concerns
**Rossi et al., 2007**[30]	Case–control, Italy	Cases n = 145 Controls n = 292	≤4 years	Yes	Yes	No apparent bias concerns
**von Linstow et al., 2008**[31]	Cohort, Denmark	N = 217	≤1 year	Yes	Yes	No apparent bias concerns
**Weber et al., 1999**[32]	Case–control, Gambia	Cases n = 277 Controls n = 364	Not specified, median 9 months	Yes	Yes	No apparent bias concerns

CHF = congestive heart failure; CLD = chronic lung disease; GA = gestational age; LRTI = lower respiratory tract infection; NR = not reported; RSV = respiratory syncytial virus; UK = United Kingdom; US = United States.

^a^ Risk status of study population: High risk = high risk of severe RSV disease; includes children born prematurely (<35 weeks gestation), of certain ethnicities (i.e. Alaska Native; Inuit), or with chronic lung disease of prematurity or congenital heart disease. Mixed risk = children with individual differences in risk of severe RSV disease; includes studies of the general population even when a minority of the study population includes high-risk children.

^b^Bias was assessed for selective reporting (not presenting all outcomes related to residential crowding) and confounding (not adjusting for other factors that may affect the association of residential crowding and severe RSV disease).

^c^ Treatment of RSV disease was not limited to the hospital setting in this study. Patients were diagnosed in the physician’s office, the emergency department, an outpatient clinic, or in multiple settings including the hospital.

^d^ In the Figueras-Aloy study of 2008 [18], one crowding variable was negative in unadjusted analysis and a second crowding variable (having school-aged siblings) was significant in the unadjusted analysis but was not entered by itself into the multivariate model.

Bias assessments for each study are presented in Table 1. Overall, there was a low risk of selective reporting or confounding across the majority of the 20 studies. Most of the studies carefully detailed the list of variables evaluated. Most studies also had a low risk of confounding bias because most reported an adjusted or multivariate analysis.

Among all the studies, crowding was measured in numerous ways. Some crowding variables assessed the number of residents per household, room, or square meter. Others measured the number or age of children or siblings in the home or how many persons shared the case child’s bed.

### Severe RSV disease—risk outcomes of residential crowding in high-risk children

Most of the studies in high-risk populations found that residential crowding significantly increased the risk of severe RSV disease as measured by at least one crowding variable (Table 2). Adjusted outcomes for crowding variables ranged from aOR 1.45 (*P* = 0.035) [15] to aOR 2.85 (*P* < 0.000001) [26].

**Table 2:** **Association of residential crowding and severe RSV disease among high-risk**^**a**^**children (N = 9)**

**Study**	**Crowding Variable**	**Unadjusted Outcome(95% CI)**	**Adjusted Outcome(95% CI)**
Broughton et al., 2005 [15]	Number of school-aged siblings	Not conducted	**aOR 1.45 (1.03–2.06),*P*** **= 0.035**
Bulkow et al., 2002 [23]	≥5 rooms in the house	*NS; OR NR*	NA
	Shares bedroom with 4 others	*NS; OR NR*	NA
	≥7 others in household	**OR 2.07 (NR),*P*** **= 0.003**	*NS, aOR NR*
	≥2 others aged <2 years in household	**OR 3.02 (NR),*P*** **= 0.011**	*NS, aOR NR*
	Household crowding index^b^ ≥2	**OR 1.71 (NR),*P*** **= 0.005**	**aOR 1.72 (NR),*P*** **= 0.024**
	≥4 others aged <12 years in household	**OR 1.91 (NR),*P*** **= 0.005**	**aOR 2.13 (NR),*P*** **= 0.011**
	Shares bed with ≥1 other person	**OR 1.74 (NR),*P*** **= 0.007**	**aOR 2.20 (NR),*P*** **= 0.036**^**c**^
Carbonell-Estrany et al., 2000 [24]	School-aged siblings (yes/no)	*P* = 0.07	**aOR 1.86 (1.01–3.4),*P*** **< 0.048**
	≥1 sibling vs. no siblings	*P* = 0.9	NA
Carbonell-Estrany et al., 2001 [25]	School-aged siblings (yes/no)	*P* = 0.01	**aOR 1.64 (1.05–2.55),*P*** **= 0.027**
Figueras-Aloy et al., 2004 [26]	≥4 inhabitants in the home excluding study subject and school-aged siblings	**OR 1.79 (1.18–2.72),*P*** **= 0.015**	**aOR 1.91 (1.19–3.07),*P =*** **0.0074**
	≥1 school-aged sibling	**OR 2.40 (1.61–3.57),*P*** **< 0.00001**	**aOR 2.85 (1.88–4.33),*P*** **< 0.000001**
Figueras-Aloy et al., 2008 [18]	≥4 inhabitants in the home excluding study subject and school-aged siblings	*NS, OR 1.37 (0.85***–***2.20), P = 0.238*	NA
	≥1 school-aged sibling	**OR 1.96 (1.47–2.60),*P*** **< 0.00001**	NA^**d**^
Kanra et al., 2005 [27]	Number of siblings	*NS*	Not conducted
Law et al., 2004 [19]	>5 people living in household including study subject	**RR 2.41 (1.42–4.06),*P*** **= 0.001**	*NS, aOR 1.69 (0.93***–***3.10), P = 0.088*
	>5 people in the household including study subject (analysis corrected for changes in risk factor status from baseline)	**OR NR*, P*** **≤ 0.002**	**aOR 1.79 (1.02–3.16),*P =*** **0.044**
	Preschool-aged siblings (yes/no)	NA	**aOR 2.76 (1.51-5.03),*P*** **= 0.001**
Simoes et al., 1993 [22]	>4 persons living in the home^e^	*RR 1.1 (0.3***–***3.9)*^c^*, P NR*	aRR NR
	>1 person per 19 m^2^ living area^e^	**RR 14.4 (1.9–109.6)**^**c**^**, (*P*** **= 0.002)**	aRR NR
	>1 child per 22 m^2^ living area^e^	**RR 8.4 (2.4–29.8)**^**c**^**, (*P*** **= 0.004)**	aRR NR
	>4 persons living in the home^f^	*NS, RR 0.77 (0.2***–***2.4)*^d^*, P NR*	*aRR NR*
	>1 person per 19 m^2^ living area^f^	*NS, RR 1.09 (0.3***–***4.5)*^d^*, P NR*	*aRR NR*
	>1 child per 22 m^2^ living area^f^	*NS, RR 2.8 (0.8***–***10.0)*^d^*, P NR*	*aRR NR*

aOR = adjusted odds ratio; aRR = adjusted relative risk; CI = confidence interval; NA = not applicable, variable not included in the model; NR = not reported; NS = not significant; OR = odds ratio; RR = relative risk; RSV = respiratory syncytial virus; UK = United Kingdom; US = United States.

Significant outcomes are presented in bold; nonsignificant outcomes are presented in italics.

^a^ High risk = high risk of severe RSV disease; includes studies of children born prematurely (<35 weeks gestation), of certain ethnicities (i.e. Alaska Natives; Inuits), or with chronic lung disease of prematurity or congenital heart disease.

^b^ Total number of persons divided by number of rooms excluding bathrooms, hallways, closets.

^c^ Multivariate outcome reported only for subgroup of children ≥6 months of age.

^d^ A joint variable, school-aged sibling(s) or day care attendance was included in the multivariate model.

^e^ Analysis based on subgroup of patients diagnosed with RSV pneumonia and hospitalized.

^f^ Analysis based on subgroup of patients diagnosed with RSV bronchiolitis; 64% were hospitalized.

Several large studies analyzed the risk factors for severe RSV disease in high-risk populations. A case–control study in Alaska natives of children <3 years of age examined numerous and varied crowding-related factors among 204 cases and 338 controls [23]. Most of the crowding variables were significantly associated with increased risk of RSV hospitalization. Most of the residential crowding variables were significant for the infants younger than 6 months compared with infants 6 months or older [23].

Among the many large epidemiological studies of RSV hospitalization conducted in Spain were populations of premature infants born at gestational ages of ≤32 weeks [24,25], 32 to 35 weeks [18], or 33 to 35 weeks [26]. In all of these studies, having one or more school-aged siblings was associated with a risk of RSV hospitalization in unadjusted [18] or adjusted [24-26] analysis. The smaller case–control study by Figueras-Aloy et al. found an association when the crowding variable was having four or more household inhabitants, not including school-aged siblings or the study subject [26]. However, this variable was not significant in the larger 2-cohort study [18].

The Pediatric Investigators Collaborative Network on Infections in Canada (PICNIC) study, which also examined children born at 32–35 weeks gestation, found that when changes in risk status following discharge from the hospital were considered, infants in homes with five or more residents were significantly at risk of hospitalization due to RSV [19]. The presence of preschool-aged siblings also was significantly associated with RSV hospitalization in both unadjusted and adjusted analyses [19].

A study in the United Kingdom examined lower respiratory tract illness due to RSV that was either diagnosed in a general practitioner’s office or resulted in hospitalization [15]. This study found an association between the number of school-aged siblings and lower respiratory tract illness due to RSV in preterm infants born at gestational ages younger than 32 weeks.

### Severe RSV disease—risk outcomes of residential crowding in mixed-risk populations of children

All of the studies in mixed-risk populations found that at least one residential crowding variable was associated with severe RSV disease in multivariate analyses (Table 3) [20,29-34]. In these studies, adjusted outcomes for crowding variables ranged from aOR 1.23 (95% confidence interval 1.01–1.56) [20] to aOR 9.1 (*P* < 0.001) [32].

**Table 3:** **Residential crowding and severe RSV disease in mixed-risk**^**a**^**study populations (N = 11)**

**Study**	**Crowding Variable**	**Unadjusted Outcome(95% CI)**	**Adjusted Outcome(95% CI)**
Albargish and Hasony, 1999 [16]	Crowding index (not defined)	*NS across 6 levels of crowding, P > 0.05*	Not conducted
Flores et al., 2004 [14]	>5 persons per household	*NS, P NR*	Not conducted
Hayes et al., 1989 [21]	Median number of children sleeping in the house	RSV-positive, 4.0 (range 2**–**11) Ill controls^b^, 3.0 (range, 1**–**18); ***P*** **= 0.005** Well controls^b^, 3.0 (range, 1–9); *NS, P NR*	Not conducted
Holberg et al., 1991 [17]	≥2 sharing room with index child	**RR 4.7 (1.6-14.2),*P*** **= 0.001**	**aRR 4.0 (1.5–10.7),*P*** **= 0.002**
	1 person sharing room with index child	*NS, RR 1.6 (0.5***–***5.5), P NR*	NA
Lanari et al., 2002 [28]	Birth order, 1–3; 4–5; ≥6	**Difference in rates of RSV positivity among children hospitalized with bronchiolitis,*P*** **< 0.05**	Not conducted
Nielsen et al.,2003 [20]	First older sibling age difference	Not conducted	**0–2 years difference, aOR 1.76 (1.45-2.32)** **2–4 years difference, aOR 1.64 (1.40-2.07)** **>4 years difference, aOR 1.23 (1.01-1.56)**
	Presence of >1 older sibling	Not conducted	*NS, aOR 1.10 (0.92***–***1.35), P NR*
	Presence of ≥1 younger sibling	Not conducted	*NS, aOR 1.02 (0.53***–***1.98), P NR*
	Square meters per resident	Not conducted	<22, *NS, aOR 1.10 (0.87-1.42) P NR* 22–28, *NS, aOR 1.14 (0.92-1.48) P NR* 28–36, *NS, aOR 1.02 (0.82-1.29) P NR* >36, Reference
Okiro et al., 2008 [9]^c^	Number of children in family	1–5, reference *6–10, NS RR 1.32 (0.85–2.04) P > 0.05* **≥11, RR 2.51 (1.32–4.76)*P*** **< 0.05**	1**–**5, reference *6****–****10, NS, aRR 0.97 (0.57****–****1.66) P > 0.05* **≥11, aRR 2.58 (1.03–6.50)*P*** **< 0.05**
	Number of siblings aged <6 years	**1–2, RR 1.78 (1.06–2.98)*P*** **< 0.05** **3–4, RR 2.00 (1.00–3.97)*P*** **< 0.05** *≥5, NS, RR 2.39 (0.81***–***7.09) P > 0.05*	**1–2, aRR 2.00 (1.17–3.42)*P*** **< 0.05** *3****–****4, NS, aRR 1.99 (0.81***–***4.91) P > 0.05* *≥5, NS, aRR 1.74 (0.54***–***5.63) P > 0.05*
Reeve et al., 2006 [29]	Presence of older sibling^d^	Not conducted	**aOR 1.6 (1.2–2.2),*P*** **= 0.005**
Rossi et al., 2007 [30]	Birth order ≥2	**OR 1.98 (1.28–3.05),*P*** **= 0.002**	**aOR 1.92 (1.21–3.06),*P*** **= 0.0049**
	≥2 children in the family	**OR 1.83 (1.16–2.88),*P*** **= 0.009**	NA^e^
von Linstow et al., 2008 [31]	Presence of older siblings	OR 3.79 (0.98–14.73), *P* = 0.054	**aOR 4.49 (1.08–18.73),*P*** **= 0.04**
Weber et al., 1999 [32]	Number of people living in the house, ≥10 vs. < 10^f^	**OR 1.59 (1.14–2.2),*P*** **= 0.006**	**aOR 3.06 (1.92–4.89),*P <*** **0.001**
	Number of children living on the compound, ≥4 vs. < 4^f^	**OR 1.7 (1.09–2.66),*P*** **= 0.02**	*NS, aOR 1.52 (0.81***–***2.85), P = 0.19*
	Number of children 2 to <3 years of age^g^	*1 child, NS, OR 1.4 (0.83–2.3), P = 0.21* *≥2 children, NS, 1.2 (0.63–2.5), P = 0.53*	**1 child, aOR 2.6 (1.2–5.6),*P =*** **0.014** *≥2 children, NS, aOR 0.78 (0.29–2.1), P = 0.63*
	Number of children 3 to 5 years of age^g^	*1 child, NS, 1.4 (0.85–2.30), P = 0.193* **≥2 children, OR 4.3 (2.4–7.8),*P*** **< 0.001**	*1 child, NS, aOR 1.9 (0.91****–****3.8), P = 0.087* **≥2 children, aOR 9.1 (3.7–23),*P*** **< 0.001**
	Siblings alive^f^, ≥3 vs. < 3	**OR 1.48 (1.05–2.09),*P*** **= 0.023**	*NS, aOR 1.17 (0.71***–***1.96)*^*b*^*, P = 0.53*

aOR = adjusted odds ratio; aRR = adjusted relative risk; CI = confidence interval; LRTI = lower respiratory tract infection; NA = not applicable, variable not included in the model; NR = not reported; NS = not significant; OR = odds ratio; RR = relative risk; RSV = respiratory syncytial virus.

Significant outcomes are presented in bold, and non-significant outcomes are presented in italics.

^a^ Mixed-risk population studies include children with individual differences in risk of severe RSV disease and studies of the general population even when a minority of the study population includes high-risk children.

^b^ In the Hayes et al. (1989) study, ill controls had lower respiratory tract illnesses that did not require hospitalization. The well controls had no lower respiratory tract illness and were not hospitalized.

^c^ Okiro et al. (2008) conducted an extensive examination of variables related to crowding. Results of unadjusted and adjusted outcomes are provided only for variables included in the adjusted analysis for the RSV–LRTI population. Variables from the unadjusted analysis were considered for inclusion in the adjusted analysis if the relevant statistical test had a *P* value of ≤0.25; for groups of collinear variables, only those with the strongest univariate association were included. Variables with statistically significant outcomes in the unadjusted model included number of family children, number of siblings aged <6 years, number of male siblings, number of siblings aged <6 years living in same house as index, number of siblings aged ≥6 years living in same house as index, number of siblings aged ≥6 years going to school. Variables with no statistically significant outcomes in the unadjusted model included number of adults sleeping in index’s room, number of siblings aged <6 years sleeping in same room as index, number of siblings aged ≥6 years sleeping in same room as index, number of siblings aged <6 years sleeping in the same bed as index, and number of siblings aged <6 years going to school.

^d^ Reported as “previous birth” or “previous pregnancy”.

^e^ Variable not entered into adjusted model because of strong autocorrelation with birth order variable.

^f^ Estimates based on 277 matched sets.

^g^ Estimates based on 172 matched sets using the extended questionnaire.

Two studies conducted in Africa examined multiple crowding-related risk factors for severe RSV disease [9,32]. In a Kenyan study, the crowding variables that were associated with RSV-related pneumonia after adjusting for other variables were the number of children in the family and the number of siblings younger than 6 years [9]. The Gambian study found that the number of people living in the house and the number of children aged 3 to 5 years significantly increased the risk of RSV hospitalization after adjusting for other factors [32].

Older siblings and the related variable birth order were associated with RSV hospitalization in studies in Australia [29], Denmark [31], and Italy [30]. A second study in Denmark of children younger than 2 years found that the presence of older or younger siblings alone was not a risk factor for RSV hospitalization, but the presence of siblings with an age difference of less than 5 years from the case child was a risk factor [20].

Of the studies in mixed-risk populations that did not report adjusted outcomes, two examined risks in hospitalized children—one in American Samoa [21] and one in Italy [28]—and two evaluated RSV disease diagnosed in settings not limited to the hospital [14,16]. In the studies of hospitalization, the Italian study found a significant risk with crowding, whereas the association was less clear in the study in American Samoa.

No associations of crowding variables were demonstrated in the two studies reporting only unadjusted outcomes for RSV disease diagnosed in the emergency department or hospital in a study in Portugal [14] or in pediatric outpatient clinics in Iraq [16]. However, a large US cohort study of healthy infants followed from birth to 12 months of age found that sharing a room with two or more persons increased the risk of RSV disease diagnosed in pediatricians’ offices even after adjusting for other factors [17].

## Discussion

The impact of environmental conditions on the risk of developing severe RSV disease is considerable once the high-risk newborn is discharged from the hospital and enters the community. Exposure to RSV is of particular concern among premature infants, who have an augmented risk of developing more severe or complicated disease owing to lower levels of maternally transmitted antibodies, reduced lung capacity for gas exchange, and increased risk of lower airways obstruction. The association between residential crowding and laboratory-confirmed RSV disease was consistent across risk status (high-risk and mixed-risk/general populations of infants), study design (cohort, case–control, prospective) and geographic location (multiple countries).

One of the major challenges of assessing residential crowding was the great variability of the study designs and descriptions of crowding, which makes the comparisons of studies difficult. The reports often assessed crowding as the number of people within a home and their proximity to the studied child, but specific definitions of crowding varied. For example, Figueras-Aloy et al. counted household residents and habitual visitors, but did not include the studied child or school-aged children [18,26]. Bulkow et al. counted the number of persons per room in the house [23], Simoes et al. [22] and Nielsen et al. [20] assessed living area per person, and Weber et al. counted the number of persons living in the compound [32].

Residential crowding can facilitate the spread of RSV infections through viral shedding under close contact conditions. Typically, maximal shedding occurs early in infection [35,36]. Children in crowded homes are more likely to be in close contact with shedders (and their secretions during that early period) and also likely to receive higher amounts of the inocula. Infants in a crowded home increase their risk of acquiring RSV at an early age. The earlier the age of acquisition, the more severe the RSV infection is as is shown by the peak age of hospitalization occurring in infants less than 3 months of age [37]. Given the heterogeneity of the studies, it is difficult to determine which residential crowding variable produces the strongest effect on RSV hospitalization. We can theorize, based on the aforementioned pathophysiology between residential crowding and RSV hospitalization that residential crowding variables such as the number and ages of others in the house, the proportion of time spent in the crowded residence (vs. daycare, etc.), and sharing the same bedroom are important considerations. Pathophysiology of residential crowding and RSV hospitalization would be very similar to the pathophysiology of daycare and RSV hospitalization where an infant is in close proximity to potentially infected children with RSV.

Further cautions should be applied to interpreting the results of this review. First, differentiating the sources of exposure that occur within versus outside the home cannot be defined with certainty. Second, the definitions and impact of crowding may be culturally determined, resulting in limited generalizability of the study’s results outside of the specific cultural setting. The 20 studies were conducted in 13 countries, including developing and industrialized countries, and, thus, one variable (e.g. number of persons per house) may have different regional impact because of variations in the typical size of homes, ventilation facilities, and climate, which may affect the intensity of exposure. For example, comparison of a study on Canadian Inuits with one of an average family in the US may be problematic; Inuits tend to live in small houses with a median of six persons per house, with limited natural air exchange, and 94% of the households include tobacco smokers [38], whereas US houses on average are larger with fewer people per room and fewer tobacco smokers. However, in most of the studies reporting adjusted analyses, residential crowding variables were significantly associated with severe RSV disease regardless of geography.

This analysis focused on children with laboratory-confirmed RSV infection. An additional analysis included studies in which the children had clinically diagnosed RSV infection or only a proportion of the children with LRTI had laboratory-confirmed RSV (data not shown). This additional analysis also supported an association between residential crowding and severe RSV disease.

Another challenge to interpreting the contribution of residential crowding as a risk factor to severe RSV disease is that the results from multivariate analyses may be confounded by inclusion of variables that closely relate to each other. For instance, residential crowding likely increases the child’s exposure to RSV, but residential crowding also can serve as a proxy for socioeconomic status. As an example, Myers and Lee found that immigrants to the US reduced their overcrowding over time as their income levels rose [39]. Thus, in some countries or regions, variables that are proxies for income could nullify or weaken the impact of residential crowding. The socioeconomic status proxies entered into the multivariate analyses in the reviewed studies included factors related to one or both parents’ education level; type of occupation of the father or major income provider; type of housing, toilet, or water sources; and status based on indices, such as postal codes. Across multiple countries and cultures, persons living in poor or less affluent areas may be subject to other factors that may affect risk of respiratory illness, such as environmental tobacco smoke and air pollutants [40,41].

## Conclusions

Despite the limitations in interpreting observational study data and the potential risk of bias in a few of the identified studies, this literature review suggests an association between crowding and laboratory-confirmed RSV disease among both high-risk and mixed-risk populations. This association was consistent despite differences in definitions of residential crowding, populations, or geographic locations.

## Abbreviations

aOR, Adjusted odds ratio; aRR, Adjusted relative risk; CHF, Congestive heart failure; CI, Confidence interval; CLD, Chronic lung disease; GA, Gestational age; LRTI, Lower respiratory tract infection; NA, Not applicable; NR, Not reported; NS, Not significant; OR, Odds ratio; RR, Relative risk; RSV, Respiratory syncytial virus; UK, United Kingdom; US, United States.

## Competing interests

This project was funded under a contract with MedImmune, LLC. Ann D. Colosia, PhD, and Amy M. Barrett, MSPH, MA, are employees of RTI Health Solutions and provided consulting services to MedImmune. Anthony Masaquel, PhD, MPH, and Parthiv J. Mahadevia, MD, MPH, are employees of MedImmune. Caroline Breese Hall, MD, and Ram Yogev, MD, were consultants on this project. There are no other competing interest disclosures.

## Authors’ contributions

A.D.C. and A.M.B. participated in the collection of data; all authors were involved with the analysis/interpretation of data and with drafting the manuscript. All authors read and approved the final manuscript.

## Pre-publication history

The pre-publication history for this paper can be accessed here:

http://www.biomedcentral.com/1471-2334/12/95/prepub

## Supplementary Material

Additional file 1: Provides search strategy to identify articles of interest from PubMed and EMBASE. Click here for file

Additional file 2: Provides full bias assessment of studies reported in this systematic literature review. Click here for file
