# Supplementary material for: Residential crowding and severe respiratory syncytial virus disease among infants and young children: A systematic literature review
Source: BMC Infect Dis. 2012 Apr 20;12:95. doi: 10.1186/1471-2334-12-95 (PMC3405464; doi:10.1186/1471-2334-12-95)
Supplement: Additional file 1: — Provides search strategy to identify articles of interest from PubMed and EMBASE. [file 1471-2334-12-95-S1.pdf]

Supplementary Table 1. PubMed Search Strategy for Studies Related to RSV and Residential Crowding or Risk Factor Analysis

Search	Search Terms
Disease MeSH Terms Limited by Subheadings and Keywords	
#1	"Respiratory Syncytial Virus Infections/complications"[Mesh] OR "Respiratory Syncytial Virus Infections/epidemiology"[Mesh] OR "Respiratory Syncytial Virus Infections/etiology"[Mesh]
#2	"Bronchiolitis, Viral/complications"[Mesh] OR "Bronchiolitis, Viral/epidemiology"[Mesh] OR "Bronchiolitis, Viral/etiology"[Mesh]
#3	"Respiratory Tract Infections/complications"[Mesh] OR "Respiratory Tract Infections/epidemiology"[Mesh] OR "Respiratory Tract Infections/etiology"[Mesh]
#4	#3 AND (syncytial OR RSV OR (acute AND lower))
#5	#1 or #2 or #4
Text Terms for Crowding	
#6	crowding[TEXT] or or resident [TEXT] or residents[TEXT] or "residential"[TEXT] or bedroom[TEXT] or bedrooms[TEXT] or household[TEXT] or households[TEXT] or "number of children"[TEXT] or "number of people"[TEXT] or "per room"[TEXT] or (> AND people) or sibling or siblings or sharing or share or "crowding"[MESH] or overcrowding
#7	#5 AND #6
Disease with Text Terms for Regression Analysis in Children	
#8	#5 and (multivariate or multifactorial or regression) and (child or children or infant or infants)
Combined Crowding and Analyses Searches	
#9	#7 or #8
Inclusionary Limits	
#8	**Limits:** Humans, English, 1985 to present
Exclusionary Limits	
#9	**Publication Type:** Editorial, Letter, Case Reports, Comment, Legal Cases, Legislation, In Vitro, Phase I clinical trial
Retained Titles/Abstracts	
#10	#8 NOT #9

Supplementary Table 2. Embase Search Strategy for Studies Related to RSV and Residential Crowding or Risk Factor Analysis

Search	Search Terms
Disease Terms	
S1	(RESPIRATORY()SYNCYTIAL()VIRUS()INFECTION? OR RESPIRATORY()SYNCYTIAL()PNEUMOVIRUS()INFECTION? OR RESPIRATORY()SYNCYTIAL()PNEUMOVIRUS/DE) AND (VIRUS()INFECTION(L)COMPLICATION OR VIRUS()INFECTION(L)EPIDEMIOLOGY OR VIRUS()INFECTION(L)ETIOLOGY)
S2	VIRAL()BRONCHIOLITIS(L)COMPLICATION OR VIRAL()BRONCHIOLITIS(L)EPIDEMIOLOGY OR VIRAL()BRONCHIOLITIS(L)ETIOLOGY
S3	RESPIRATORY TRACT INFECTION!(L)COMPLICATION OR RESPIRATORY TRACT INFECTION!(L)EPIDEMIOLOGY OR RESPIRATORY TRACT INFECTION!(L)ETIOLOGY
S4	S3 AND (SYNCYTIAL OR RSV OR (ACUTE AND LOWER))
S5	S1 OR S2 OR S4
Terms for Crowding	
S6	CROWDING OR RESIDENT OR RESIDENTS OR RESIDENTIAL OR BEDROOM OR BEDROOMS OR HOUSEHOLD OR HOUSEHOLDS OR NUMBER(1W)CHILDREN OR NUMBER(1W)PEOPLE OR PER()ROOM OR (">" AND PEOPLE) OR SIBLING OR SIBLINGS OR SHARING OR SHARE OR CROWDING/DE OR OVERCROWDING
S7	S5 AND S6
Disease with Terms for Regression Analysis in Children	
S8	S5 AND (MULTIVARIATE OR MULTIFACTORIAL OR REGRESSION) AND (CHILD OR CHILDREN OR INFANT OR INFANTS)
Combined Crowding and Analyses Searches	
S9	S7 OR S8
Inclusionary Limits	
S10	S9/HUMAN
S11	S10/ENG
S12	S11/1985:2009
Exclusionary Limits	
S13	S12 NOT (DT=EDITORIAL OR DT=LETTER OR CASE()REPORT/DE OR COMMENT?/TI OR LEGAL()CASE? OR LAW/DE OR IN VITRO STUDY! OR PHASE()1()CLINICAL()TRIAL/DE)
Retained Titles/Abstracts	
S14	RD S13 (unique items)
