# Supplementary material for: Residential crowding and severe respiratory syncytial virus disease among infants and young children: A systematic literature review
Source: BMC Infect Dis. 2012 Apr 20;12:95. doi: 10.1186/1471-2334-12-95 (PMC3405464; doi:10.1186/1471-2334-12-95)
Supplement: Additional file 2: — Provides full bias assessment of studies reported in this systematic literature review. [file 1471-2334-12-95-S2.pdf]

Risk of Bias* Related to Residential Crowding and Serious RSV Disease					
		Free of selective reporting?		Free of confounding bias?	
Study type	Reference	Judgement		Judgement	Description
Cohort (of children with ARI)	Albargish KA, Hasony HJ. Respiratory syncytial virus infection among young children with acute respiratory tract infection in Iraq. East Mediterr Health J 1999 Sep;5:941-8.	Low risk	Unadjusted analysis fully reported	High risk	No multivariate analysis to address confounding
Cohort	Broughton S, Roberts A, Fox G, Pollina E, Zuckerman M, Chaudhry S, Greenough A: Prospective study of healthcare utilisation and respiratory morbidity due to RSV infection in prematurely born infants. Thorax 2005, 60(12):1039-1044.	Low risk	Multivariate analysis fully reported	Low risk	Conducted multivariate analysis to address confounding
Case-control	Bulkow LR, Singleton RJ, Karron RA, et al. Risk factors for severe respiratory syncytial virus infection among Alaska native children. Pediatrics 2002; 109:210-6.	Low risk	Multivariate analysis fully reported	Low risk	Conducted multivariate analysis to address confounding
Cohort	Carbonell-Estrany X, Quero J, Bustos G, Cotero A, Domenech E, Figueras-Aloy J, Fraga JM, Garcia LG, Garcia-Alix A, Del Rio MG et al: Rehospitalization because of respiratory syncytial virus infection in premature infants younger than 33 weeks of gestation: a prospective study. IRIS Study Group. Pediatr Infect Dis J 2000, 19(7):592-597.	Low risk	Multivariate analysis fully reported	Low risk	Conducted multivariate analysis to address confounding
Cohort	Carbonell-Estrany X, Quero J: Hospitalization rates for respiratory syncytial virus infection in premature infants born during two consecutive seasons. Pediatr Infect Dis J 2001, 20(9):874-879.	Low risk	Multivariate analysis fully reported	Low risk	Conducted multivariate analysis to address confounding

Study type	Reference	Free of selective reporting? Judgement		Free of confounding bias? Judgement	Description
Case-control	Figueras-Aloy J, Carbonell-Estrany X, Quero J: Case-control study of the risk factors linked to respiratory syncytial virus infection requiring hospitalization in premature infants born at a gestational age of 33-35 weeks in Spain. Pediatr Infect Dis J 2004, 23(9):815-820.	Low risk	Multivariate analysis fully reported	Low risk	Conducted multivariate analysis to address confounding
Cohort (2-cohort)	Figueras-Aloy J, Carbonell-Estrany X, Quero-Jimenez J, Fernandez-Colomer B, Guzman-Cabanas J, Echaniz-Urcelay I, Domenech-Martinez E: FLIP-2 Study: risk factors linked to respiratory syncytial virus infection requiring hospitalization in premature infants born in Spain at a gestational age of 32 to 35 weeks. Pediatr Infect Dis J 2008, 27(9):788-793.	Low risk	Multivariate analysis fully reported	Low risk	Conducted multivariate analysis to address confounding
Cohort	Flores P, Rebelo-de-Andrade H, Gonçalves P, et al. Bronchiolitis caused by respiratory syncytial virus in an area of Portugal: epidemiology, clinical features, and risk factors. Eur J Clin Microbiol Infect Dis 2004;23:39-45.	Low risk	Unadjusted analysis fully reported	High risk	No multivariate analysis to address confounding
Case-control	Hayes EB, Hurwitz ES, Schonberger LB, Anderson LJ: Respiratory syncytial virus outbreak on American Samoa. Evaluation of risk factors. Am J Dis Child 1989, 143(3):316-321.	Low risk	Unadjusted analysis fully reported	High risk	No multivariate analysis to address confounding
Cohort	Holberg CJ, Wright AL, Martinez FD, Ray CG, Taussig LM, Lebowitz MD: Risk factors for respiratory syncytial virus-associated lower respiratory illnesses in the first year of life. Am J Epidemiol 1991, 133(11):1135-1151.	Low risk	Multivariate analysis fully reported	Low risk	Conducted multivariate analysis to address confounding

Study type	Reference	Free of selective reporting?		Free of confounding bias?	
		Judgement		Judgement	Description
Cohort	Kanra G, Tezcan S, Yilmaz G. Respiratory syncytial virus epidemiology in Turkey. Turk J Pediatr 2005;47:303-8.	Low risk	Unadjusted analysis fully reported	High risk	No multivariate analysis to address confounding
Cohort	Lanari M, Giovannini M, Giuffre L, et al. Prevalence of respiratory syncytial virus infection in Italian infants hospitalized for acute lower respiratory tract infections, and association between respiratory syncytial virus infection risk factors and disease severity. Pediatr Pulmonol 2002;33:458-65.	High risk	Unadjusted analysis not fully reported	High risk	No multivariate analysis to address confounding
Cohort	Law BJ, Langley JM, Allen U, et al. The Pediatric Investigators Collaborative Network on Infections in Canada study of predictors of hospitalization for respiratory syncytial virus infection for infants born at 33 through 35 completed weeks of gestation. Pediatr Infect Dis J 2004; 23:806-14.	Low risk	All risk factors significant in bivariate analysis are listed in Table 4, and all risk factors significant in multivariate analysis are listed in Table 5.	Low risk	Conducted multivariate analysis to address confounding
Case-control	Nielsen HE, Siersma V, Andersen S, et al. Respiratory syncytial virus infection--risk factors for hospital admission: a case-control study. Acta Paediatr 2003;92:1314-21.	Low risk	Multivariate analysis full reported	Low risk	Conducted multivariate analysis to address confounding
Cohort	Okiro EA, Ngama M, Bett A, et al. Factors associated with increased risk of progression to respiratory syncytial virus-associated pneumonia in young Kenyan children. Trop Med Int Health 2008;13:914-26.	Low risk	Multivariate analysis fully reported	Low risk	Conducted multivariate analysis to address confounding

Study type	Reference	Free of selective reporting?		Free of confounding bias?	
		Judgement		Judgement	Description
Case-control	Reeve CA, Whitehall JS, Buettner PG, et al. Predicting respiratory syncytial virus hospitalisation in Australian children. J Paediatr Child Health 2006;42:248-52.	Low risk	Multivariate analysis full reported	Low risk	Conducted multivariate analysis to address confounding
Case-control	Rossi GA, Medici MC, Arcangeletti MC, et al. Risk factors for severe RSV-induced lower respiratory tract infection over four consecutive epidemics. Eur J Pediatr 2007;166:1267-72.	Low risk	Adjusted analysis fully reported	Low risk	Conducted multivariate analysis to address confounding
Case-control	Simoes EA, King SJ, Lehr MV, et al. Preterm twins and triplets. A high-risk group for severe respiratory syncytial virus infection. Am J Dis Child 1993;147:303-6.	High risk	Multivariate analysis not fully reported	High risk	No multivariate analysis outcomes to address confounding was reported for crowding variables
Cohort	von Linstow ML, Hogh M, Nordbo SA, Eugen-Olsen J, Koch A, Hogh B: A community study of clinical traits and risk factors for human metapneumovirus and respiratory syncytial virus infection during the first year of life. Eur J Pediatr 2008, 167(10):1125-1133.	Low risk	Multivariate analysis fully reported	Low risk	Conducted multivariate analysis to address confounding
Case-control	Weber MW, Milligan P, Hilton S, et al. Risk factors for severe respiratory syncytial virus infection leading to hospital admission in children in the western region of the Gambia. Int J Epidemiol 1999;28:157-62.	Low risk	Multivariate analysis fully reported	Low risk	Conducted multivariate analysis to address confounding

a Risk of bias related to adequate sequence generation, allocation concealment, blinding of participants and personnel, or blinding of outcome assessment is not relevant to noninterventional studies such as the ones included in this literature review. Assessments for these types of bias have not been included as all would be "not applicable."
